# An approach of state-of-charge prediction for lithium-ion batteries using hybrid Pyraformer-LSTM learning network

**DOI:** 10.1371/journal.pone.0353298

**Published:** 2026-07-24

**Authors:** Xianbin Wang, Liping Zhang, Qihua Fan, Yiyin Hu, Jian Zhang, Huimin Chen

**Affiliations:** 1 Institute of General Aviation Industry, Fujian Chuanzheng Communications College, Fuzhou, China; 2 School of Aerospace Engineering, Xiamen University, Xiamen, China; 3 School of Aeronautics, Changji University, Changji, China; Sunway University, MALAYSIA

## Abstract

Accurate prediction of the State of Charge (SOC) of lithium-ion batteries remains difficult under complex operating conditions. This difficulty arises from strong nonlinearity and long-term temporal dependency in battery data. In this study, we developed a hybrid prediction model that combines a pyramidal attention network with a Long Short-Term Memory (LSTM) network. The key innovation of this work lies in the integration of a pyramidal attention mechanism with a multi-scale sliding window design, enabling hierarchical extraction of temporal features ranging from local dynamic variations to long-term evolutionary trends. Specifically, the pyramidal attention module captures multi-granularity temporal dependencies through dynamic weighted fusion of features from different window scales (8, 16, and 32), while the LSTM network captures nonlinear dynamics and preserves long-term dependencies through gated temporal modeling. We evaluated the proposed method using battery datasets collected under multiple temperatures. We compared its performance with six representative SOC estimation methods. The experimental results show that the proposed model achieves RMSE and MAE of 1.8554% and 1.3021% on the test set, significantly outperforming baselines, demonstrating high prediction accuracy and validating the effectiveness of multi-scale attention and recurrent structures in SOC prediction.

## 1. Introduction

In recent years, rapid global industrial growth has led to a continuous increase in energy demand [1]. Under the dual pressures of rising carbon emissions and declining fossil fuel reserves, the energy supply gap has been steadily widening, thereby accelerating the progress of the energy transition [2]. In fields such as new energy vehicles, drones, and personal mobile devices, lithium-ion batteries have become an indispensable core energy unit due to their high energy density, fast charging capability, relatively low cost, and low environmental impact [3]. They serve as a crucial technological foundation for modern energy storage and conversion systems [4]. As application scenarios continue to expand and operating conditions become increasingly complex, ensuring the safe and efficient operation of battery packs through a Battery Management System (BMS) has emerged as a critical challenge [5,6]. Against this backdrop, precise management and real-time monitoring of battery states have become increasingly important. In particular, accurate prediction of the State of Charge (SOC) of lithium-ion batteries is a fundamental prerequisite for ensuring safe operation, optimizing performance, and extending battery lifespan [7,8].

The prediction of the SOC of lithium-ion batteries is a core technology within BMS. According to the definition provided by the U.S. Advanced Battery Consortium [9], SOC represents the percentage of a battery’s remaining capacity relative to its rated capacity at a given discharge rate, indicating the available energy. Current research on SOC prediction exhibits a mixed development pattern, encompassing both traditional approaches and emerging data-driven techniques [10,11].

Early methods for estimating the SOC of lithium-ion batteries primarily relied on experimental approaches, such as the ampere-hour counting method [12] and the Open Circuit Voltage (OCV) method [13]. These techniques are fundamentally based on establishing a mapping between SOC and externally measurable battery characteristics. The ampere-hour counting method estimates SOC by integrating the current over time. It offers the advantages of simplicity and ease of implementation. However, it is highly dependent on the initial SOC and is prone to error accumulation from current measurements, which limits its real-time performance and robustness [14]. To address these limitations, Li et al. [15] proposed an improved ampere-hour counting method that incorporates capacity fade and efficiency correction, which enhances the estimation accuracy to a certain extent. The OCV method estimates SOC by constructing an OCV–SOC lookup table. However, this approach requires the battery to remain idle for extended periods, and the OCV–SOC relationship is sensitive to factors such as temperature and aging, which limits its practical applicability in engineering [16,17].

With the advancement of model-based approaches, equivalent circuit models, such as the Thevenin model [18] and partnership for a new generation of vehicles model [19], combined with Kalman filtering algorithms, have been widely used for SOC estimation under dynamic operating conditions [20]. By employing state-space modeling and parameter identification, these methods achieve both high computational efficiency and strong interpretability. Nevertheless, their accuracy strongly relies on model fidelity, and they struggle to represent battery aging and time-varying behaviors precisely [21]. For instance, Guo et al. [22] proposed an improved unscented Kalman filter to enhance linearization accuracy while reducing computational complexity. However, real-world noise often violates ideal assumptions, which can still lead to convergence difficulties.

In recent years, with the advancement of intelligent algorithms and computational capabilities, data-driven approaches have gradually become a research focus for SOC estimation [23]. Support Vector Machine (SVM) employs kernel functions to realize nonlinear mappings and exhibits strong performance in small-sample scenarios [24]. Li et al. [25] applied SVM to SOC prediction and achieved promising results. However, its training complexity and limited scalability restrict its applicability to large-scale datasets. In contrast, neural networks have demonstrated greater potential for SOC estimation due to their strong nonlinear representation capability. Recurrent Neural Network (RNN) and its variants, including Long Short-Term Memory (LSTM) network and Transformer, are capable of effectively modeling temporal dependencies in voltage, current, and other battery time-series signals [26,27]. Hu et al. [28] employed a Bayesian-optimized TCN-LSTM approach for lithium-ion battery SOC estimation, achieving high prediction accuracy. Tian et al. [29] developed an attention-driven CONV-LSTM model that combines CNN and LSTM to capture the spatiotemporal dependencies of battery data, achieving more accurate and stable SOC prediction. Chen et al. [30] developed an LSTM network with extended inputs and constrained outputs to enhance nonlinear feature modeling. Furthermore, Zhou et al. [31] developed an attention-enhanced LSTM model with hyperparameters optimized via grid search, enabling the extraction of critical information and improving SOC estimation accuracy under highly nonlinear and variable operating conditions.

Recently, to address the challenges of model complexity and hyperparameter tuning in neural network-based methods, Bayesian optimization has been employed to optimize network architectures for SOC estimation. Wang et al. [32] proposed a BO-BiLSTM-UKF fusion algorithm that combines bidirectional LSTM with Bayesian hyperparameter optimization and unscented Kalman filtering for noise correction, achieving high-precision SOC prediction with strong robustness. Furthermore, anti-noise adaptive mechanisms have been explored to enhance model stability under noisy operating conditions. Wang et al. [33] developed an ANA-LSTM network with multi-feature collaboration and adaptive feedback correction, demonstrating improved robustness for battery state prediction. Beyond purely data-driven approaches, hybrid model-data driven methods have also gained attention. Wang et al. [34] introduced a multi-feature electrochemical-thermal coupling model combined with a decoupled deviation-extended Kalman filter for accurate SOC estimation under low-temperature conditions.

Despite significant advances in SOC estimation achieved by the aforementioned neural network–based methods, certain limitations remain under complex operating conditions. Existing single-architecture models, such as standalone LSTM or Transformer variants, often struggle to simultaneously capture both local dynamic variations and long-term evolutionary trends. For instance, LSTM-based approaches primarily rely on recursive mechanisms that constrain their ability to model global dependencies in long time series, while pure attention-based models may lack sufficient inductive bias for sequential temporal smoothing. These limitations reduce the robustness and generalization of SOC prediction under complex and nonstationary conditions.

To address the aforementioned limitations, this study proposes a Pyraformer-LSTM hybrid framework for lithium-ion battery SOC prediction. The proposed method leverages a pyramidal attention mechanism to extract multi-scale temporal features through hierarchical windowed self-attention, enabling efficient modeling of both short-term dynamics and long-range dependencies in battery time series. These learned multi-scale representations are subsequently refined by an LSTM network, which applies gated sequential processing to capture nonlinear battery dynamics and preserve temporal continuity. By integrating the complementary strengths of attention-based feature extraction and LSTM-based sequence modeling, this hybrid architecture addresses the limitations of conventional single-architecture approaches. The main contributions of this paper are summarized as follows:

(i) A Pyraformer-LSTM hybrid modeling framework for lithium-ion battery SOC prediction is proposed. In the encoding stage, the pyramidal attention structure of Pyraformer is incorporated to hierarchically and multi-scale model the input time series, enabling temporal features ranging from local dynamic variations to long-term evolutionary trends to be systematically captured.
(ii) Within this framework, the multi-scale features encoded by the Pyraformer are fused into an LSTM network, fully leveraging LSTM’s strength in nonlinear temporal modeling. This enables both short- and long-term dependencies to be jointly captured, overcoming the limitations of a single LSTM in representing complex temporal features.
(iii) A series of systematic experiments were conducted under multiple temperature conditions to benchmark the proposed method against six representative SOC estimation algorithms. Comprehensive evaluations across prediction accuracy, stability, and generalization demonstrate the effectiveness and superiority of the Pyraformer-LSTM model. The results indicate that the hybrid framework not only significantly enhances SOC prediction accuracy but also validates the necessity and rationality of integrating Pyraformer with LSTM for collaborative temporal feature modeling.

## 2. The proposed model

**Fig 1:**
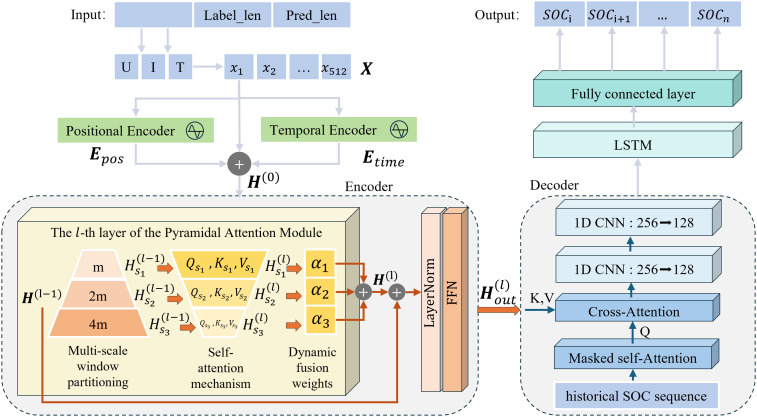
Architecture of the proposed Pyraformer-LSTM model.

The overall architecture of the proposed model is illustrated in Fig 1. The model takes voltage, current, and temperature as inputs and outputs the predicted SOC. The encoder adopts the Pyraformer framework with a pyramidal multi-scale attention mechanism to hierarchically model the input time series and capture short-, mid-, and long-term dependencies across multiple temporal resolutions. These multi-scale representations are dynamically fused via learnable weights, enabling effective temporal feature extraction with reduced computational complexity. The decoder combines a cross-attention layer with convolutional modules to leverage global multi-scale temporal representations while enhancing the modeling of local dynamic variations. An LSTM module is subsequently employed to refine the prediction sequence through gated temporal smoothing, thereby improving prediction stability and accuracy by preserving long-term dependencies.

### 2.1 Pyramidal attention-based encoder-decoder architecture

The encoder takes a historical time series $𝐗=\{x_{\text{1}},x_{\text{2}},\ldots ,x_{T}\},\text{\textbackslash{}hspace\{1em}\}x_{t}\in {\mathbb{R}}^{d}$ of length T as input. To enhance temporal representation, positional encoding and temporal encoding are incorporated to inject chronological order and temporal priors into the input. Positional encoding models the absolute and relative ordering of time steps, while temporal encoding captures periodic patterns and time-interval characteristics. The resulting temporally enriched representation is formulated as follows:


$$\begin{array}{c} {𝐇}^{\left(0\right)}=𝐗+{𝐄}_{pos}+{𝐄}_{time} \end{array}$$ (1)

where ${𝐄}_{pos}$ denotes the positional encoding term, ${𝐄}_{time}$ represents the temporal encoding term, and ${𝐇}^{\left(0\right)}$ corresponds to the initial input representation of the encoder.

The embedded sequence features are passed through 3 stacked pyramidal attention layers for hierarchical modeling. Each layer employs 8 attention heads with head dimension of 64. The feedforward network within each layer expands the dimension to 2048 followed by GELU activation. Residual connections and layer normalization are applied after both the attention and feedforward sub-layers to stabilize training. In the l*-th* layer, the input is denoted as $H^{\left(1-1\right)}$. A multi-scale sliding-window mechanism is adopted to model local temporal dependencies in parallel across different time scales. Given a set of window sizes $𝒮=\{s_{\text{1}},s_{\text{2}},\ldots ,s_{k}\}$ with $s_{k}\in \left\{8,16,32\right\}$, the sequence is partitioned into non-overlapping or slightly overlapping subsequences, within which local self-attention is computed independently. For the window at scale $s_{k}$, the corresponding local feature representation is denoted as ${𝐇}_{s_{k}}^{\left(l-1\right)}\in {\mathbb{R}}^{s_{k}\times d_{\text{model}}}$. Within each window, local self-attention is computed independently. Under this mechanism, the representation of any time step *t* depends only on its neighboring time steps within the same window, thereby avoiding redundant modeling of distant and irrelevant nodes. This design significantly reduces computational complexity while enhancing the ability to capture local dynamic patterns.

After computing local attention across different window scales, multi-scale feature representations $\left\{{𝐇}_{s_{\text{1}}}^{\left(l\right)},{𝐇}_{s_{\text{2}}}^{\left(l\right)},\ldots ,{𝐇}_{s_{K}}^{\left(l\right)}\right\}$ are obtained. These multi-level temporal features are then adaptively aggregated through a dynamic weighted fusion layer.


$$\begin{array}{c} {𝐇}^{\left(l\right)}=\sum_{k=1}^{K}\alpha_{k}{𝐇}_{s_{k}}^{\left(l\right)} \end{array}$$ (2)

$$\begin{array}{c} \alpha_{k}=\frac{exp\left({𝐰}_{k}^{⊤}{𝐇}_{s_{k}}^{\left(l\right)}\right)}{\sum_{j=1}^{K}exp\left({𝐰}_{j}^{⊤}{𝐇}_{s_{j}}^{\left(l\right)}\right)} \end{array}$$ (3)

where $\alpha_{k}$ denotes the dynamic weight for each temporal scale, adaptively adjusting the contribution of features at different scales. This mechanism enables the construction of a hierarchical pyramidal representation, capturing information from local to global patterns.

From a structural perspective, the window sizes define the temporal span of the pyramidal attention, while the number of pyramid layers governs the depth of hierarchical temporal abstraction. This design allows the model to simultaneously capture short-term local fluctuations and long-term evolutionary trends. To improve training stability and enhance representational capacity, each pyramid module integrates residual connections and normalization, as expressed by the following formulation:


$$\begin{array}{c} {\tilde{𝐇}}^{\left(l\right)}=\text{LayerNorm}\left({𝐇}^{\left(l\right)}+{𝐇}^{\left(l-1\right)}\right) \end{array}$$ (4)

The multi-scale features are further processed through a feedforward neural network to perform nonlinear feature transformations. This step enhances interactions and fusion among different temporal scales while preserving the underlying sequential information.


$$\begin{array}{c} {𝐇}_{\text{out}}^{\left(l\right)}=\text{FFN}\left({\tilde{𝐇}}^{\left(l\right)}\right) \end{array}$$ (5)

The decoder is designed hierarchically to progressively enhance its ability to model temporal dependencies in the target sequence. It first applies masked self-attention to the historical SOC sequence, enforcing causality to align with the temporal evolution of SOC and ensuring that predictions rely solely on known past information. Subsequently, a standard cross-attention layer uses the masked self-attention output as the query to interact with the encoder’s global multi-scale temporal features, effectively capturing contextual relationships between input variables and SOC dynamics and strengthening global predictive modeling. To further enhance local feature extraction, a convolutional module is applied to the cross-attention output, refining the representation of dynamic changes between adjacent time steps and compensating for the limitations of pure attention mechanisms in capturing local patterns. By combining cross-attention with convolution, the decoder fully leverages the encoder’s multi-scale representations while precisely modeling local dynamics. The decoder employs two convolutional layers with kernel size 3 and GELU activation to progressively reduce the feature dimension from 512 to 256 and then to 128, enhancing local feature extraction before LSTM processing.

The encoder-decoder module produces a sequence of high-level temporal representations that encode both multi-scale structural patterns and local dynamic variations. These representations serve as the bridge between Pyraformer’s feature extraction capability and LSTM’s sequential refinement strength. The encoder’s pyramidal attention identifies temporal correlations at multiple scales, while the decoder’s cross-attention aligns these features with SOC evolution dynamics. However, the resulting representations may still exhibit local oscillations inherent to attention mechanisms. To address this, the LSTM module is introduced to perform gated temporal smoothing, ensuring that predictions are not only accurate but also temporally consistent.

### 2.2 LSTM-based prediction layer

In the prediction stage, the model applies an LSTM layer to the high-level temporal features generated by the decoder for further sequential refinement. The motivation for incorporating LSTM after the Pyraformer encoder-decoder lies in the complementary nature of attention-based feature extraction and recurrent temporal modeling.

While the Pyraformer encoder-decoder effectively captures multi-scale temporal patterns through hierarchical attention, the resulting representations may lack the explicit sequential inductive bias that recurrent networks provide. LSTM addresses this limitation through its gating mechanism, which enables adaptive filtering of noisy temporal features while preserving important long-term dependencies. Specifically, the input gate controls how much new information enters the cell state, the forget gate determines what historical information to retain or discard, and the output gate regulates the final hidden state used for prediction. This gated architecture naturally produces temporally smooth predictions, reducing the local oscillations that pure attention mechanisms may generate.

The LSTM outputs are then passed through a fully connected layer to project the high-dimensional temporal representations onto the target prediction space, enabling accurate regression of SOC values. This design ensures that the multi-scale features extracted by Pyraformer are further refined into smooth and temporally consistent predictions, combining the strengths of both architectures.

### 2.3 Loss function

The loss function serves as a fundamental metric for quantifying the discrepancy between predicted and true values, playing a critical role in model training. It guides the learning process by providing gradients for parameter updates, enabling the model to fit the data effectively. Beyond training, the loss function also functions as an essential evaluation metric for assessing model performance and comparing alternative models. Careful selection of an appropriate loss function can mitigate overfitting and enhance the model’s generalization capability.

This study considers Mean Squared Error (MSE), Mean Absolute Error (MAE), and the Huber loss as candidate loss functions for model training and performance evaluation. MSE measures predictive accuracy by averaging the squared differences between predicted and true values. The squaring operation assigns greater penalty to larger errors, making MSE particularly sensitive to outliers and significant deviations, thereby encouraging the model to reduce substantial prediction errors. Let $y_{i}$ and ${\hat{y}}_{i}$ denote the true and predicted SOC values, respectively. MSE is defined as follows:


$$\begin{array}{c} MSE=\frac{\text{1}}{n}\sum_{i=1}^{n}(y_{i}-{\hat{y}}_{i}{)}^{\text{2}} \end{array}$$ (6)

MAE computes the average of the absolute differences between predicted and true values, quantifying the model’s overall average deviation. Unlike MSE, MAE is less sensitive to outliers, providing a more robust measure of typical prediction errors. It is formally defined as follows:


$$\begin{array}{c} MAE=\frac{\text{1}}{n}\sum_{i=1}^{n}\left|y_{i}-{\hat{y}}_{i}\right| \end{array}$$ (7)

The Huber loss offers a compromise between MSE and MAE by combining their advantages. For small prediction errors, it behaves like MSE, applying a squared penalty to maintain high sensitivity and enable fine-grained fitting. When errors exceed a predefined threshold, it transitions to an MAE-like linear penalty, effectively limiting the influence of outliers or noisy samples on model training. This dual behavior allows the Huber loss to balance predictive accuracy with robustness, capturing minor deviations precisely while preventing large errors from dominating gradient updates. Such properties make it particularly suitable for SOC prediction under noisy or highly variable operating conditions. The Huber loss is mathematically defined as follows:


$$\begin{array}{c} Huber\left(y_{i},{\hat{y}}_{i}\right)=\left\{ \begin{array}{c} @l@\frac{\text{1}}{\text{2}}(y_{i}-{\hat{y}}_{i}{)}^{\text{2}},\;\;\left|y_{i}-{\hat{y}}_{i}\right|\leq \delta \;\\ \delta \cdot (\left|y_{i}-{\hat{y}}_{i}\right|-\frac{\text{1}}{\text{2}}\delta ),\;\;\left|y_{i}-{\hat{y}}_{i}\right|>\delta \; \end{array}\right. \end{array}$$ (8)

The prediction errors of the model on the test set under different loss function configurations are summarized in Table 1. As shown, training with MSE yields the lowest RMSE and MAE values, indicating the best overall predictive performance. Based on this comparative analysis, MSE is selected as the loss function for all subsequent experiments and performance evaluations.

**Table 1:** Comparison of model prediction errors using three different loss functions.

Loss Functions	RMSE (%)	MAE (%)
MSE	1.8554	1.3021
MAE	1.9051	1.3689
Huber	1.9732	1.4429

To provide a clear overview of the practical implementation, the training and prediction workflow of the proposed model is summarized in the form of pseudocode. The process begins with data loading and batch partitioning, followed by iterative execution of multi-scale feature encoding, temporal modeling, feature fusion, and SOC prediction in each training epoch. Prediction errors are computed using MSE loss, and model parameters are updated via backpropagation. This procedure is repeated across batches until the predefined number of epochs is reached or convergence criteria are satisfied, offering an intuitive representation of the model’s overall operational logic.

**Algorithm 1. Training Procedure of the Pyraformer-LSTM Model**

**Input:** Historical time series samples 𝐗, corresponding historical SOC labels 𝐘, pyramidal window scale set $\left\{w_{\text{1}},w_{\text{2}},\ldots ,w_{S}\right\}$, number of training epochs $N_{\text{epoch}}$, learning rate lr.

**Output:** Trained model parameters Θ.

1 Initialize model parameters Θ.

2 **for** epoch = 1 to $N_{\text{epoch}}$ **do**

**3**  Sample a batch of data $\left({𝐗}_{b},{𝐘}_{b}\right)$ from the training set.

4  Encoder stage: feature embedding and multi-scale modeling.

5  Inject positional encoding and temporal encoding into the input sequence to obtain the initial representation.

6  **for** each pyramidal attention layer l=1 to *L* **do**

**7**   **for** $w_{\text{i}}$ to $\left\{w_{\text{1}},w_{\text{2}},\ldots ,w_{S}\right\}$ **do**

**8**     Partition the sequence according to the window size and perform local self-attention.

**9**   **end for**

**10**     Dynamically fuse features from different scales using learnable weights.

**11**     Apply a feedforward neural network for nonlinear feature transformation.

**12** **end for**

**13**  Obtain the encoder output features ${𝐇}_{\text{enc}}$.

**14**  Decoder stage: temporal alignment and context modeling to produce decoder output representations ${𝐇}_{\text{dec}}$.

**15**  Prediction stage: SOC regression.

**16**  Feed the decoder output into the LSTM network for sequential refinement.

**17**  Map the LSTM output to the SOC prediction space through a fully connected layer.

**18**  Obtain the predicted SOC values ${𝐘}_{b}$.

**19**  Compute the MSE loss.

**20**  Backpropagate to update model parameters Θ.

**21 end for**

## 3. Experiments

### 3.1 Dataset selection and preprocessing

#### 3.1.1 CLACE dataset.

**Fig 2:**
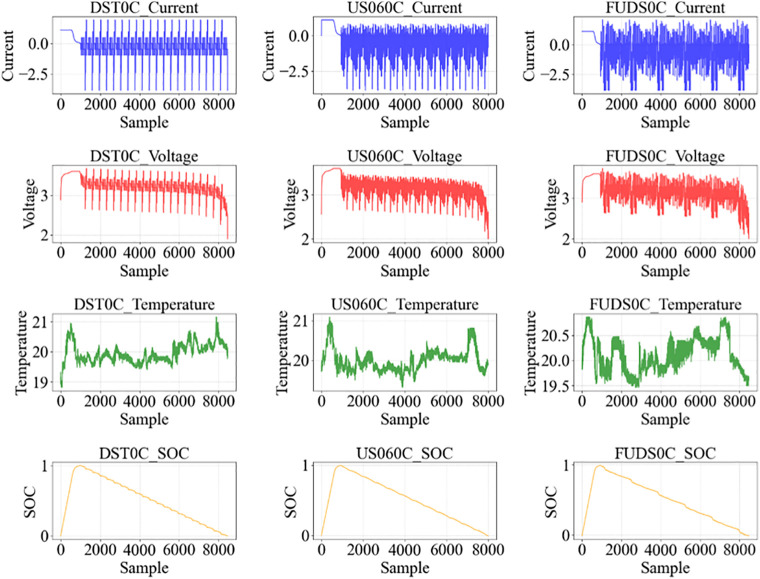
Current, voltage, temperature, and SOC profiles under the three driving cycles at 0 °C.

In this study, we select the A123 subset of the CALCE dataset, which contains multi-feature lithium-ion battery data under varying temperatures and operating conditions. Data from the US06 and DST driving cycles are used as the training set, while data from the FUDS cycle serve as the test set, enabling evaluation of both prediction accuracy across diverse operating scenarios and the robustness to different driving patterns. Fig 2 illustrates the voltage, current, temperature, and SOC profiles under the three driving cycles. Notably, the DST cycle exhibits pronounced pulse currents and sharp voltage spikes, though the variations in voltage and current remain relatively simple compared to real-world charge-discharge conditions. In contrast, the US06 cycle, simulating highway driving conditions, produces the most severe fluctuations with the highest variation frequency due to rapid voltage ramps and complex current dynamics. The FUDS cycle, representing urban stop-and-go driving, features frequent but small-amplitude fluctuations. These diverse and complex operating conditions present significant challenges for SOC prediction algorithms, providing a rigorous test of their robustness and accuracy.

#### 3.1.2 Data preprocessing.

In the CALCE dataset, the A123 cells were first fully discharged to establish a baseline, ensuring that the residual capacity at the start of subsequent tests was zero. Following full discharge, the cells underwent simulated charge-discharge cycles under three distinct driving profiles. For discrete measurements, the fully discharged points within each cycle were identified, and the cumulative charge during each charging process was computed. The maximum cumulative charge was then taken as the current cell capacity, which serves as the reference for SOC calculation. SOC values were subsequently determined using a discrete formulation based on these cumulative charge measurements, providing a consistent and systematic method to quantify the battery’s available capacity across different cycles.


$$\begin{array}{c} SOC\left(k\right)=SOC(k-1)-\frac{\Delta t}{Q_{n}}I(k-1) \end{array}$$ (9)

where k denotes the k-th time step, and Δt represents the time interval between the k-th and (k−1)-th steps.

To mitigate the effects of varying scales and magnitudes among the raw features and to facilitate faster convergence and improved prediction accuracy, the voltage, current, and temperature data were standardized prior to model training. The standardization process transforms each feature to have zero mean and unit variance, as defined by the following equation:


$$\begin{array}{c} z=\frac{x-\mu }{\sigma } \end{array}$$ (10)

where x denotes the original feature value, μ represents the mean of the feature in the training set, σ is the corresponding standard deviation, and z denotes the standardized feature representation. By standardizing the data in this manner, the model benefits from faster convergence during training and enhanced robustness in its predictions.

### 3.2 Experimental settings

In this study, the experimental parameters are consistently configured to ensure training stability and reliable prediction performance. The model takes fixed-length time series of 96 samples as input, which enables sufficient characterization of the dynamic behavior of lithium-ion batteries during continuous operation. In the feature extraction stage, a pyramidal attention mechanism is employed with multi-scale window sizes set to 8, 16, and 32, allowing for the joint modeling of local temporal details and global trend information across different time scales.

For model training, the learning rate is set to $10^{-4}$ to balance convergence speed and training stability. A dropout rate of 0.05 is applied to mitigate overfitting and enhance generalization capability. The MSE is adopted as the loss function to quantify the discrepancy between the predicted and true SOC values, thereby guiding parameter updates during optimization. These hyperparameter settings are determined through extensive empirical validation and achieve a favorable trade-off between stable convergence and prediction accuracy.

### 3.3 Analysis of prediction results

To evaluate the predictive performance and accuracy of the proposed Pyraformer-LSTM algorithm for lithium-ion battery SOC estimation, this study utilizes the A123 battery dataset from the CALCE database under various operating conditions and temperature settings. Specifically, data from the DST and US06 driving cycles are employed for model training, while data from the FUDS driving cycle are used for testing. A systematic analysis of SOC prediction performance is conducted at five temperature levels: 0°C, 10°C, 20°C, 30°C, and 40°C.

**Fig 3:**
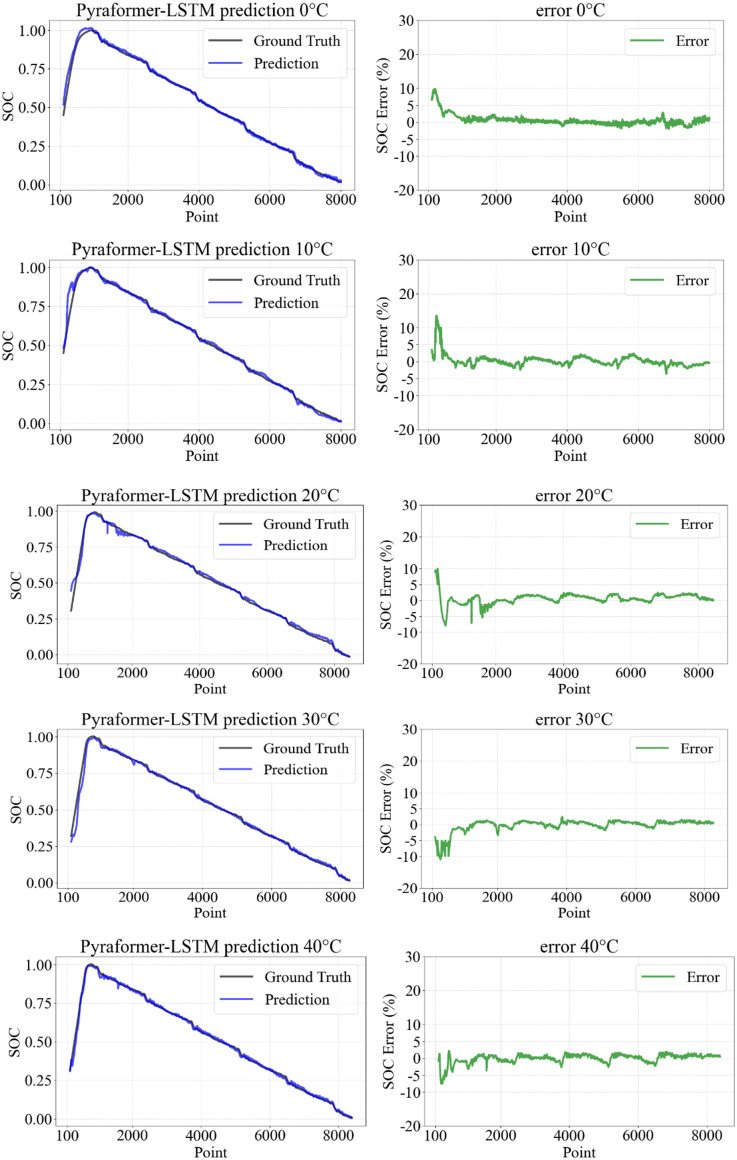
SOC prediction curves of the Pyraformer–LSTM model at different temperatures (from top to bottom: 0°C, 10°C, 20°C, 30°C, and 40°C).

To quantitatively assess the prediction performance under different temperature conditions, the MAE and Root Mean Square Error (RMSE) are adopted as evaluation metrics. The RMSE and MAE results at each temperature are presented in Table 2, and the corresponding SOC prediction curves are illustrated in Fig 3.

**Table 2:** Prediction errors of the Pyraformer–LSTM algorithm.

Temperature	RMSE(%)	MAE(%)
0°C	**1.8554**	1.3021
10°C	2.5634	1.4266
20°C	2.5325	1.4606
30°C	2.3395	1.3085
40°C	1.8955	**1.1913**

The results summarized in Table 2 demonstrate that the proposed Pyraformer-LSTM framework consistently achieves superior performance compared with the previously developed Informer-LSTM model incorporating a global-local attention mechanism. Notably, higher estimation accuracy is observed at 0°C, 30°C, and 40°C, which is consistent with earlier findings. The improved performance at 30°C and 40°C can be attributed to the relatively stable electrochemical kinetics under ambient and moderately elevated temperatures, leading to more regular dynamic characteristics that can be more effectively captured by the learning model. Overall, these findings highlight the pronounced impact of temperature variation on the accuracy of lithium-ion battery SOC estimation. Notably, the consistent RMSE ranging from 1.86% to 2.56% across all temperature conditions demonstrates the model’s robustness to temperature variations when evaluated on the unseen FUDS driving cycle.

Fig 3 presents the SOC prediction results under different temperature conditions. The best discharge-phase accuracy is achieved at 0°C, which may be attributed to the greater use of 0°C data during hyperparameter tuning, enhancing model adaptability to this condition. However, the overall variation in error metrics across temperatures is marginal, demonstrating robust cross-temperature generalization.

In contrast, at 10°C and 20°C, the charging-phase predictions are less accurate than those during discharging. This is likely due to data imbalance, as charging samples constitute less than 13% of the dataset, limiting the model’s ability to capture charging dynamics. Overall, the proposed Pyraformer-LSTM model delivers stable and accurate SOC estimation across all evaluated temperature conditions.

To further investigate model performance, the discharge segments were separately evaluated, and the corresponding MAE and RMSE values are reported in Table 3. The results indicate that the prediction accuracy during discharging is substantially higher than the overall performance, with errors reduced by approximately one order of magnitude, particularly under low-temperature conditions.

**Table 3:** Prediction errors of the discharge phase using the Pyraformer-LSTM Model.

Temperature	RMSE(%)	MAE(%)
0°C	**0.7330**	**0.5718**
10°C	0.9754	0.8117
20°C	1.0425	0.9040
30°C	1.0823	1.0674
40°C	1.0520	0.8829

**Fig 4:**
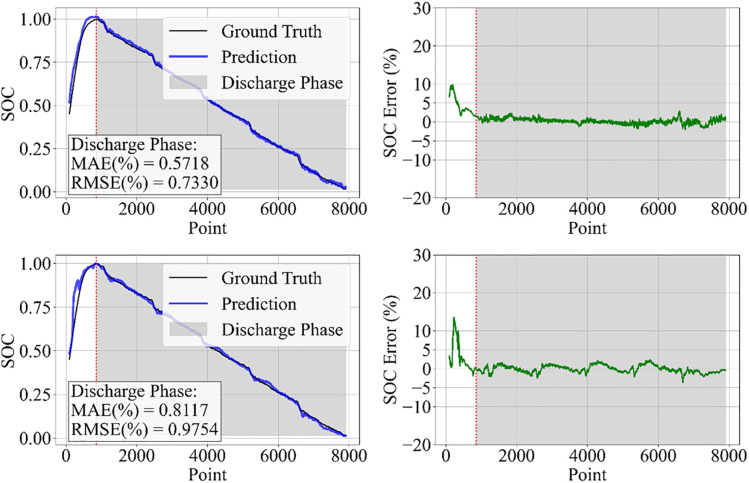
Illustration of the discharge segment in the predicted curve.

The discharge-phase prediction curves are illustrated in Fig 4, where the shaded regions denote the discharging intervals. The predicted trajectories closely align with the ground truth in both global trends and local dynamics, with deviations consistently maintained within 5% and fluctuating around zero. This demonstrates the model’s strong capability in capturing discharge characteristics. In contrast, the charging-phase accuracy is relatively lower, likely due to data imbalance, as charging samples account for less than 13% of the dataset, limiting sufficient feature learning for this regime. Despite this imbalance, the proposed Pyraformer-LSTM model maintains stable and reliable SOC estimation performance across all temperature conditions.

### 3.4 Ablation study

An ablation study is conducted to systematically investigate the roles of the key components in the proposed Pyraformer-LSTM model and to quantify their contributions to overall performance. Specifically, the experiments focus on the pyramidal attention module, the LSTM unit, and their integration strategy. By selectively removing, replacing, or modifying these components, the resulting performance variations in time-series prediction are analyzed. This experimental design provides clear empirical evidence of the functional importance of each module, thereby offering valuable insights for structural optimization and guiding the development of a more efficient and robust SOC prediction framework.

In the experiments, representative samples from the A123 battery subset of the CALCE dataset are selected to construct and evaluate three models: a Pyraformer model incorporating only the pyramidal attention mechanism, an LSTM model relying solely on temporal sequence modeling, and the proposed hybrid Pyraformer-LSTM model that integrates both components. SOC prediction is performed under five temperature conditions (0 °C, 10 °C, 20 °C, 30 °C, and 40 °C), and the prediction errors of each model are computed for comparative analysis. The corresponding results are summarized in Table 4. As can be observed, the Pyraformer-LSTM consistently outperforms the standalone Pyraformer and LSTM models across all temperature scenarios, achieving the lowest errors on both evaluation metrics, which confirms the effectiveness of the proposed hybrid architecture.

**Table 4:** Prediction errors of Pyraformer, LSTM, and Pyraformer-LSTM.

Temperature	Model	RMSE(%)	MAE(%)
0°C	Pyraformer-LSTM	**1.8554**	**1.3021**
	Pyraformer	2.6395	1.7485
	LSTM	6.5290	4.4756
10°C	Pyraformer-LSTM	**2.5634**	**1.4266**
	Pyraformer	2.7233	2.2701
	LSTM	4.9368	4.5646
20°C	Pyraformer-LSTM	**2.5325**	**1.4606**
	Pyraformer	2.8718	1.8855
	LSTM	6.8903	4.5390
30°C	Pyraformer-LSTM	**2.3395**	**1.3085**
	Pyraformer	2.5234	2.2903
	LSTM	4.7169	4.5847
40°C	Pyraformer-LSTM	**1.8955**	**1.1913**
	Pyraformer	2.5595	1.7486
	LSTM	4.5235	4.4326

**Fig 5:**
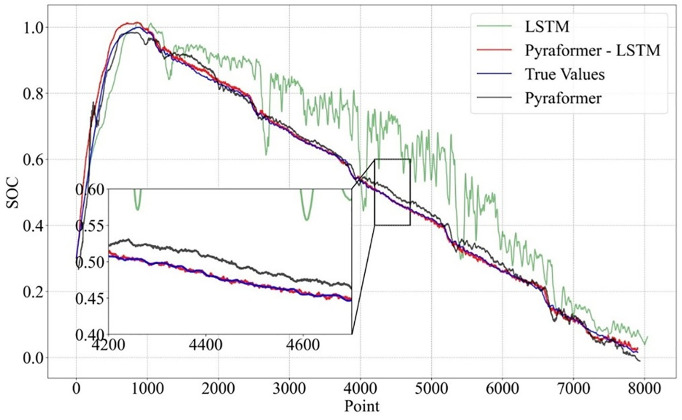
Prediction curves of the three algorithms at 0 °C.

To further examine the predictive behavior of different models, the SOC prediction trajectories and corresponding error curves on the test set under the 0 °C condition are plotted for all three methods in Fig 5. The left panel presents the overall prediction results, while the right panel provides a magnified view of the highlighted region.

From the global curves, it can be observed that the standalone LSTM model exhibits pronounced fluctuations, with substantial deviations from the true SOC during the discharge phase. This occurs because LSTM has limited capability in modeling short-term fluctuations, which causes the predicted curve to fail to accurately track the real SOC curve. While LSTM can capture the global trend, its overreaction to local fluctuations results in substantial errors.

The Pyraformer-only model achieves a lower overall error than LSTM and shows improved alignment with the ground truth. However, noticeable local oscillations remain, indicating room for improvement in smoothness and consistency. The Pyraformer model, leveraging the pyramid multi-scale attention mechanism, captures features at different time scales through its multi-level attention structure. This allows Pyraformer to effectively capture trend changes over long time spans. As a result, its overall prediction accuracy is significantly improved compared to LSTM. However, some local fluctuations still remain, and the model’s smoothness and fitting consistency could be further refined. This limitation arises because Pyraformer is efficient in capturing global trends. Nevertheless, it may overlook local features, especially during rapid SOC changes. As a result, prediction errors occur in certain localized regions.

In contrast, the proposed Pyraformer-LSTM demonstrates superior performance in both global trend tracking and local dynamic representation, maintaining a high level of smoothness and agreement with the true SOC throughout the entire time horizon. This advantage becomes more evident in the zoomed-in view, where the Pyraformer-LSTM predictions closely overlap with the reference SOC and accurately capture subtle variations. The Pyraformer-LSTM hybrid model combines the multi-scale attention mechanism of Pyraformer with the temporal modeling capabilities of LSTM. The multi-scale features extracted by Pyraformer provide rich temporal information to LSTM, enabling it to better capture both short-term and long-term dependencies. With the support of Pyraformer, LSTM no longer relies solely on its limited modeling capability but utilizes Pyraformer’s global information to improve predictions of local fluctuations. Therefore, the hybrid model outperforms both the individual Pyraformer and LSTM models in capturing both local dynamics and global trends. This enhances the overall accuracy and stability of predictions. The results of the ablation study strongly validate the effectiveness and necessity of combining LSTM and Pyraformer for improving SOC prediction accuracy and stability.

### 3.5 Comparative experiments

To validate the superiority of the proposed Pyraformer-LSTM framework, a comprehensive comparison was conducted using several mainstream data-driven algorithms on the A123 battery subset of the CALCE dataset. All models, including GRU, LSTM, Pyraformer, Informer, Informer-LSTM [35], and LGInformer-LSTM with global-local attention, were trained and evaluated under identical conditions. SOC predictions were generated, corresponding performance metrics were computed, and prediction curves were plotted to facilitate direct comparison. This experimental setup enables a systematic assessment of the relative performance, robustness, and predictive accuracy of the Pyraformer-LSTM model against established baselines.

The average prediction errors of all algorithms across five temperature conditions (0 °C, 10 °C, 20 °C, 30 °C, and 40 °C) are summarized in Table 5. The results clearly demonstrate that the proposed Pyraformer-LSTM consistently achieves the best predictive performance across all temperatures, with overall error levels significantly lower than those of the competing methods, indicating superior stability and adaptability. Among the comparison models, the LGInformer-LSTM incorporating global-local attention and the standalone Pyraformer also deliver relatively strong performance, yet both exhibit noticeable fluctuations across different temperature scenarios. In contrast, Pyraformer-LSTM maintains consistently high prediction accuracy under all temperature conditions, further confirming its effectiveness and robustness for SOC estimation in complex, multi-temperature operating environments.

**Table 5:** Average prediction errors of each algorithm.

Model	RMSE(%)	MAE(%)
Pyraformer-LSTM	**2.23726**	**1.33782**
LGInformer-LSTM	2.7446	1.8793
Informer-LSTM	3.2273	2.0836
Pyraformer	2.6635	1.9886
Informer	4.0865	2.5329
GRU	4.2467	2.4132
LSTM	5.5193	4.5193

**Fig 6:**
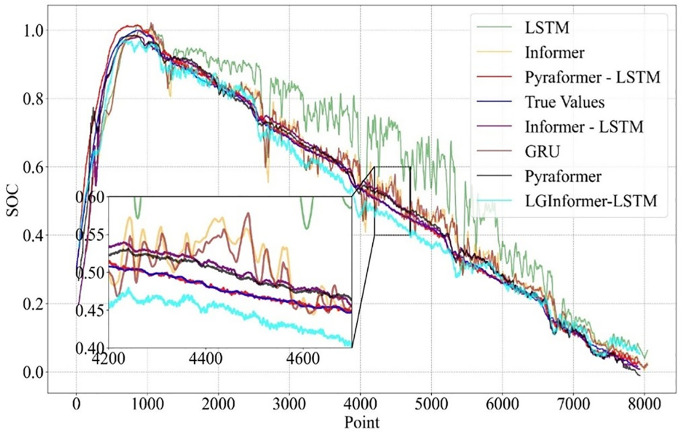
SOC prediction curves of all algorithms under 0 °C conditions.

**Fig 7:**
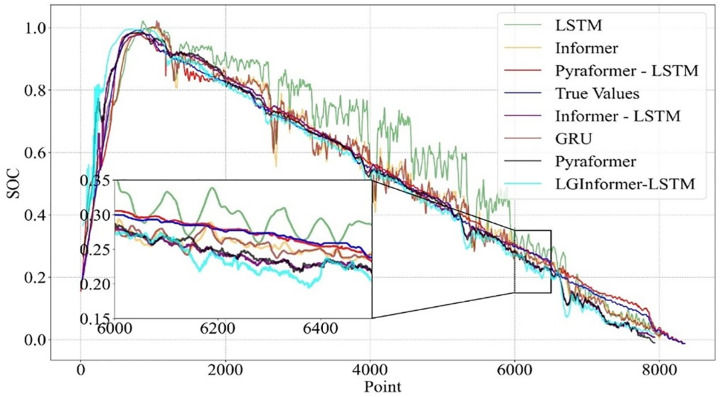
SOC prediction curves of all algorithms under 20 °C conditions.

To further evaluate the models’ fitting capabilities in practical SOC prediction tasks, the predicted SOC curves of seven models under 0 °C and 20 °C conditions were aggregated, with representative discharge segments magnified for detailed inspection, as shown in Figs 6 and 7. Across both global trends and local variations, our model’s predictions closely match the true SOC values, demonstrating superior fitting performance and minimal prediction fluctuations. The standalone Pyraformer also captures the discharge dynamics effectively, benefiting from its pyramidal attention mechanism. The LGInformer–LSTM, incorporating global-local attention, achieves moderate accuracy, with relatively small deviations in the magnified local segments, yet still falls slightly short of the overall performance of Pyraformer-LSTM. Notably, traditional models such as LSTM and GRU exhibit larger discrepancies in capturing local details, often showing lag or excessive oscillations, highlighting their limited capacity to model complex time-series under extreme conditions. By integrating multi-scale pyramidal attention with LSTM units, the Pyraformer-LSTM model effectively extracts dynamic features across different temporal scales while preserving long-term dependencies, resulting in substantially improved SOC prediction accuracy.

## 4. Conclusions

This study proposes a novel Pyraformer-LSTM hybrid framework for lithium-ion battery SOC prediction. The key innovations of this work are threefold. To begin with, we introduce a pyramidal multi-scale attention mechanism into the SOC estimation task, which enables hierarchical feature extraction across different temporal resolutions. Unlike conventional LSTM models that rely solely on recursive temporal modeling, our pyramidal attention structure can simultaneously capture local dynamic variations, mid-term trends, and long-range dependencies through its multi-scale sliding-window design. Moreover, we design a stage-wise collaboration strategy between Pyraformer and LSTM, where Pyraformer handles multi-scale feature extraction and the LSTM module performs sequential refinement through gated temporal smoothing. This complementary architecture addresses the limitation of single-structure models in representing complex temporal features. Finally, we integrate cross-attention and convolutional modules in the decoder to align Pyraformer features with SOC dynamics while enhancing local pattern modeling, further improving prediction accuracy.

Given the pronounced impact of temperature on battery SOC prediction, experiments are conducted on the CALCE dataset, which provides lithium-ion battery operating data under five distinct temperature conditions. Under identical experimental settings, six representative data-driven models are constructed for comparative evaluation, and each model is tested separately at the five temperature levels to assess adaptability and robustness. The experimental results demonstrate that the proposed Pyraformer-LSTM model consistently achieves the lowest RMSE and MAE across all temperature scenarios, significantly outperforming the baseline methods. These findings demonstrate the effectiveness of combining pyramidal multi-scale attention with LSTM-based sequential modeling for accurate and robust SOC estimation under multi-temperature operating conditions evaluated on the CALCE dataset.
